# Mud Pack With Menthol and *Arnica Montana* Accelerates Recovery Following a High-Volume Resistance Training Session for Lower Body in Trained Men

**DOI:** 10.1519/JSC.0000000000003751

**Published:** 2020-09-17

**Authors:** Sandro Bartolomei, Federico Nigro, Alessio D'Amico, Matteo Cortesi, Rocco Di Michele

**Affiliations:** 1 Department of Biomedical and Neuoromotor Sciences, University of Bologna, Bologna, Italy; and; 2 Department for Life Quality Studies, University of Bologna, Bologna, Italy

**Keywords:** strength, muscle morphology, muscle soreness

## Abstract

Bartolomei, S, Nigro, F, D'Amico, A, Cortesi, M, and Di Michele, R. Mud pack with menthol and *Arnica montana* accelerates recovery following a high-volume resistance training session for lower body in trained men. *J Strength Cond Res* 36(7): 1909–1915, 2022—The aim of this study was to compare the effects of a mud pack, containing menthol and *Arnica montana*, on the recovery responses following a high-volume resistance protocol. Ten resistance-trained men (mean ± *SD*: age = 25.3 ± 6.1 years; body mass = 79.1 ± 10.6 kg; height = 178.9 ± 7.5 cm) performed a high-volume resistance workout for lower body squat and leg extension, (5 sets of 10 reps at 70% of one repetition maximum for both exercises). All the subject used mud (MUD) or a placebo (PL) in randomized counterbalanced crossover design. MUD or PL were applied 4 times: 3, 19, 27, and 45 hours after the workout, on the skin surface above the quadriceps muscle of both legs. Muscle performance (countermovement jump power [CMJP], isokinetic leg press at 75 cm·s^−1^ and 25 cm·s^−1^ [ISOK75 and ISOK25, respectively], isometric squat [ISQ]), and morphology (muscle thickness of vastus lateralis [VLMT]), were measured before exercise (baseline [BL]), and 15 minutes (15P), 24 hours (24P), and 48 hours (48P) postexercise. In addition, muscle soreness was assessed at the same time points using a visual analog scale (VAS). No significant interactions (*p* > 0.05) between the trials were detected for CMJP, ISOK75, ISQ, and VLMT. A significant interaction between trials was noted for ISOK25 (*p* = 0.022) and for VAS (*p* = 0.001). ISOK25 was significantly (*p* < 0.01) reduced from BL at 15P, 24P and 48P in PL, whereas changes were significant (*p* < 0.05) at 15P and 24P only in MUD. The present findings indicate that MUD may enhance the recovery rate of strength and reduce muscle soreness after high-volume exercise. Muscle morphology do not seem to be influenced by mud packs.

## Introduction

Recovery after training sessions and competitive events represents a key factor in optimizing training adaptations to obtain optimal performance. Many topical products and recovery strategies are used by athletes in the attempt of enhancing the recovery process and to reduce the perception of muscle soreness following exercise (6). Some commercial topical products used by athletes after exercise or during the warm-up contain menthol as an active ingredient. Menthol is a cyclic terpene alcohol (16) that has been shown to promote vasoconstriction of blood vessels (22,32) and subjective feeling of coolness (11). A reduction in blood flow through the radial artery induced by the application of a 3.5% menthol gel to the forearm was reported by Topp et al. (32). The effect was attributed to short-acting neuronal mechanisms of sympathetic reflex induced by menthol (33). In addition, menthol has been demonstrated to inhibit TRPA1 channels that mediate inflammation and pain (25), and to activate k-opioid receptors inducing an increase in pain threshold (15).

A study by Tokunaga et al. (31), reported increased neuromuscular activation in lower body muscles when a 5% menthol gel was applied on the skin over the working muscles. Recently, significant positive effects of menthol gels were reported, when applied during the recovery phase following sprint training (16). In this study, the topical application of a 4% menthol gel enhanced the recovery rate of lower-body power produced during the vertical jump.

Mud pack therapy has been used as an effective alternative treatment for rheumatic musculoskeletal pain since time immemorial (13,14). Mud is a mixture of organic and inorganic matter with water, resulting from a natural process with the influence of several physicochemical factors (8). The beneficial effect of mud pack treatment for chronic diseases such as osteoarthritis, has been often ascribed to heat conduction (8). Some studies however, suggest that anti-inflammatory and antiseptic effects of mud may be also related to inorganic components that are adsorbed through the skin and may increase electric conductance and absorption (18). This study (18) also demonstrated relevant anti-inflammatory effects of mud packs, reducing serum levels of IL-1, YKL-40, and prostaglandin E2. A study by Obasasi et al. (27) reported enhanced anti-inflammatory effects when mud was applied directly to the skin, compared with nylon-covered mud, in patients affected by osteoarthritis. In addition, other studies reported significant reductions in osteoarticular pain with mud therapy at ambient temperature. The effects may be related to a ionic exchange between the mud and the body through the skin, induced by the presence of iron, copper, and chromium (18). Despite the relevant effects of mud packs on reducing inflammation, no information exists about the effect of mud, including menthol and *Arnica montana* on resistance exercise-induced muscle damage. Extracts from this plant have been historically used for its anti-inflammatory properties (1). The effect of *Arnica montana* has been attributed to the presence of helenaline, a sesquiterpene lactone (24). In vitro and animal studies reported relevant anti-inflammatory effects of this drug and inhibitory effects on the transcriptional nuclear factor kB (24). However, no studies to date reported significant effects of topical *Arnica* on exercise-induced muscle pain (1). Despite the fact that thermal mud therapy is typically performed using warm mud without additional components, many athletes and sport enthusiasts use commercial products at ambient temperature, made by mud and essential oils or plant extracts to relieve joint or muscle pain. Thus, the aim of this study was to evaluate the effects of mud packs, including menthol and *Arnica montana*, on the recovery response following a high-volume resistance training protocol for lower body. Previous research (2) indeed, have reported significant muscle damage associated with similar high-volume resistance exercise protocols. It was hypothesized that mud packs may reduce exercise-induced muscle soreness following a high-volume exercise session for lower body.

## Methods

### Experimental Approach to the Problem

**Figure 1.**
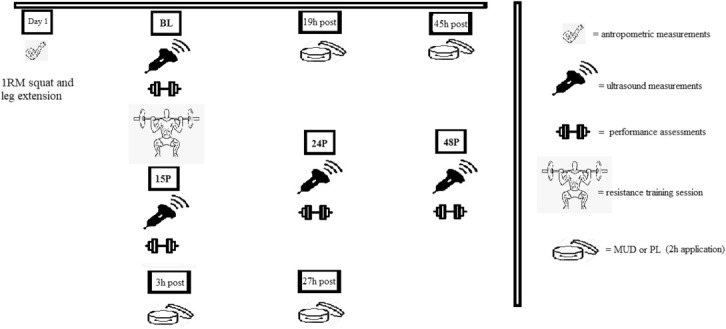
Timeline of the experimental design followed by the subject in MUD and PL trial.

The experimental protocol consisted of a counterbalanced cross-over research design (Figure 1). The subject were requested to visit the laboratory on 9 separate occasions. During the first visit, subject were assessed for one repetition maximum strength (1RM) at the squat and leg extension exercises. In addition, they performed a selection of lower body maximal isometric force and power assessments. Baseline (BL) anthropometric measures were also determined. Subject reported back to the laboratory at least 72 hours after their initial visit and were randomly assigned into either an exercise protocol using menthol and *Arnica montana* mud packs (MUD) or into a placebo (PL) protocol. Subsequently, they performed a high-volume resistance training workout, and were tested 15 minutes postexercise (15P) to assess the acute fatiguing effect of the exercise. All the subject were asked to self-apply MUD or PL on the skin for 2 , 3, 19, 27, and 45 hours after the workout. Subject reported back to the laboratory 24 hours (24P) and 48 hours (48P) postexercise for the subsequent assessments.

Ultrasonography was obtained at each time point (BL, 15P, 24P, and 48P). After at least 10 days of rest (average 14.4 ± 6.1 days) from the end of the first testing session, subject reported back to the laboratory and performed the opposite protocol.

### Subjects

Ten experienced, resistance-trained men (mean ± *SD* age: 25.3 ± 6.1 years; body mass: 79.1 ± 10.6 kg; height: 178.9 ± 7.5 cm; body fat: 13.5 ± 3.4%, resistance training experience: 8.2 ± 4.2 years; squat 1RM: 141.9 ± 26.0 kg) volunteered to participate in this study. Inclusion criteria were age 18–35 years, a minimum of 2 years of resistance training experience, and ability to squat at least 1.5 times their body mass. Subject were not permitted to use any additional dietary supplementation, and did not consume any androgens or other performance enhancing drugs. Screening for performance enhancing drug use and additional supplementation was accomplished with a questionnaire completed at the recruitment stage. The study was approved by the University of Bologna's institutional review board. Testing procedures were fully explained to each subject before obtaining individual written informed consent.

### Procedures

#### Strength and Power Testing

Before testing, subject performed a standardized warm-up consisting of 5-minute cycling on a cycle ergometer against a light resistance, 10 body weight squats, 10 body weight walking lunges, 10 dynamic walking hamstring stretches, and 10 dynamic walking quadriceps stretches (4). The 1RM test for the barbell back squat was performed using methods previously described by Hoffman (20). Each subject was asked to perform 2 warm-up sets using a resistance of approximately 40–60 and 60–80% of his perceived maximum, respectively. For each exercise, 3–4 subsequent trials were performed to determine the 1RM. A 3–5-minute rest period was provided between each trial. Trials not meeting the appropriate range of motion criteria for each exercise, or with incorrect lifting technique, were discarded. During all other visits, the same standardized warm-up, was repeated. During each visit, subject were required to perform a countermovement jump (CMJ) on a contact mat (Globus Ergo Jump, Globus Inc. Codognè, Italy). Subject were instructed to maximize the height of each jump while keeping the hands on their hips. Flight time was calculated as the time interval from toe-off to landing. The jump height was estimated as 9.81 × flight time^2^/8 (7). Peak power (CMJ power [CMJP], expressed in W) was calculated by the jump height and the subject's body mass using the following equation (29):

Peak Power= 60.7× jump height+45.3×body mass−2055

Subject performed 3 jumps with a 3-minute rest between each jump. The intraclass coefficient calculated for the CMJP in the present study was 0.96 (*SEM* = 122.0 W).

Isometric and isokinetic strength measurements were performed following the CMJP on a linear isokinetic dynamometer (LIDO Loredan Leg Press). Subject were seated and stabilized to the device, with the right leg attached to the lever arm. Isokinetic concentric measurements were obtained by performing 2 maximal unilateral extensions of the lower limb at 75 cm·s^−1^ (ISOK75) and at 25 cm·s^−1^ (ISOK25). A 3-minute rest was provided between the trials. The highest peak force value was recorded. Intraclass coefficients were 0.90 (*SEM* = 37.8 N) and 0.92 (*SEM* = 39.5 N) for ISOK75 and ISOK25, respectively. An isometric parallel squat (ISQ) assessment was performed using a power rack that permitted fixation of the bar at a different height while the subject stood on a force plate (Kistler 9,260, 500hz, Winterthur, Switzerland). Subject were required to assume a parallel squat position, with hips and knees at the same level, and to perform 2 maximal 6-second isometric contractions with a 3-minute recovery time between each attempt. Knee and hip angles were measured using a goniometer to reproduce the same position for all testing sessions and peak force was measured. Intraclass coefficients were 0.85 (*SEM* = 822.5 N·s^−1^) for ISQ. During all isometric and isokinetic measurements, subject were verbally encouraged to perform at maximum effort.

#### Ultrasonography Measurements

Noninvasive skeletal muscle ultrasound images were collected from the subject's right thigh as a measurement of muscle swelling. Before image collection, all anatomical locations of interest were identified using standardized landmarks for the vastus lateralis (VL muscle). The landmark for the VL was identified along its longitudinal distance at 50% from the proximal insertion of the muscle. The length of the VL encompassed the distance from the lateral condyle of the tibia to the most prominent point of the greater trochanter of the femur. Vastus lateralis measurement required the subject to lay on their side on the examination table for a minimum of 15 minutes before images were collected. The same investigator performed all landmark measurements for each subject. A 12-MHz linear probe scanning head (Echo Wave 2, Telemed Ultrasound Medical System, Milan, Italy) was coated with water soluble transmission gel to optimize spatial resolution and used to collect all ultrasound images. The probe was positioned on the surface of the skin without depressing the dermal layer and the view mode (gain = 50 dB; image depth = 5 cm) was used to take panoramic pictures of the VL. During the measurements, subject were asked to relax their leg muscles and maintain the left lateral decubitus position. Legs were positioned together, with a 10° bend angle in the knees (5). All ultrasound images were taken and analyzed by the same technician. Muscle thickness (MT) measures were obtained using a longitudinal B-mode image. Three consecutive MT images were captured and analyzed for each muscle and leg, respectively. For each image, MT was measured with a single perpendicular line from the superficial aponeurosis to the deep aponeurosis. The average of the 3 MT measures was used for statistical analyses. Intraclass correlation coefficients (ICCs) for VLMT was 0.92 (*SEM* = 0.10 cm).

#### Muscle Soreness Score

Subject were instructed to assess their subjective feelings of soreness intensity using a 100-mm visual analog scale (VAS) (26). No soreness was recorded as 0 and the worst possible soreness as 100. Soreness was evaluated at BL, 15P, 24P and 48P.

#### Resistance Workout Protocol

The high-volume resistance training workout included the squat and leg extension exercises. The subject performed 5 sets of 10 repetitions at 70% of the previously measured 1RM in both exercises. Recovery time between sets was 75 seconds. A 2-second time, was used during the eccentric and concentric phases. Time was kept with a stopwatch and seconds were counted out loud by an investigator. If the required number of repetitions per set was not completed, the load was reduced in the subsequent set to enable the subject to complete the required number of repetitions in that set. No forced repetitions were performed. All training sessions were supervised by the same investigator (certified strength coach). Training was performed at the same time of the day (2 pm) throughout the study to avoid any influence of diurnal variation of performance.

#### MUD Pack Applications

Subject were required to self-apply MUD or PL 3, 19, 27, and 45 hours after the exercise session and to keep the product on the skin for 2 hours in each application (Figure 1). Subject were instructed to apply the product on the external surface above both quadriceps muscles without external pressure or massage. Premeasured amounts of products were given to each subject to maintain consistency in the product applications. The following equation was used to determine the amount of MUD or PL to obtain approximatively the same product concentration in different individuals:

MUD or PL(g) per leg=individual body mass(kg)/1.7

MUD used in the present investigation consisted of a solid argillaceous component, predominantly inorganic with a 5% of menthol, and a 3% of *Arnica montana*. PL consisted of natural moisturizing cream similar in color and consistency to MUD. Because of the absence of menthol or camphor in the PL, however, the products did not smell the same and differed in the cooling effect on the skin surface. Both products were applied on the skin surface at ambient temperature.

### Statistical Analysis

All data are presented as mean ± *SD*. For all the examined variables, Shapiro-Wilk tests were used to assess the normal distribution of the data. If the assumption of sphericity was violated, a Greenhouse-Geisser correction was applied.

Performance and ultrasound data were analyzed using a 2-factor (trial × time) analysis of variance (ANOVA) with repeated measures. In the event of a significant F ratio, dependent *t* tests with a Bonferroni adjustment were used to examine pairwise comparison between trials at each time point. In the event of a significant trial × time interaction, each group was analyzed separately by a one-factor ANOVA with repeated measures on time. Partial eta squared were reported as the effect size (ES), and were interpreted according to Stevens (30), with 0.01, 0.06, and 0.14 representing small, medium, and large ESs, respectively. Where appropriate, percent change was calculated as follows: [(post-exercise mean−pre-exercise mean)/pre-exercise mean] × 100. Significance was set at an alpha level of *p* ≤ 0.05.

## Results

### Performance Assessments

**Figure 2.**
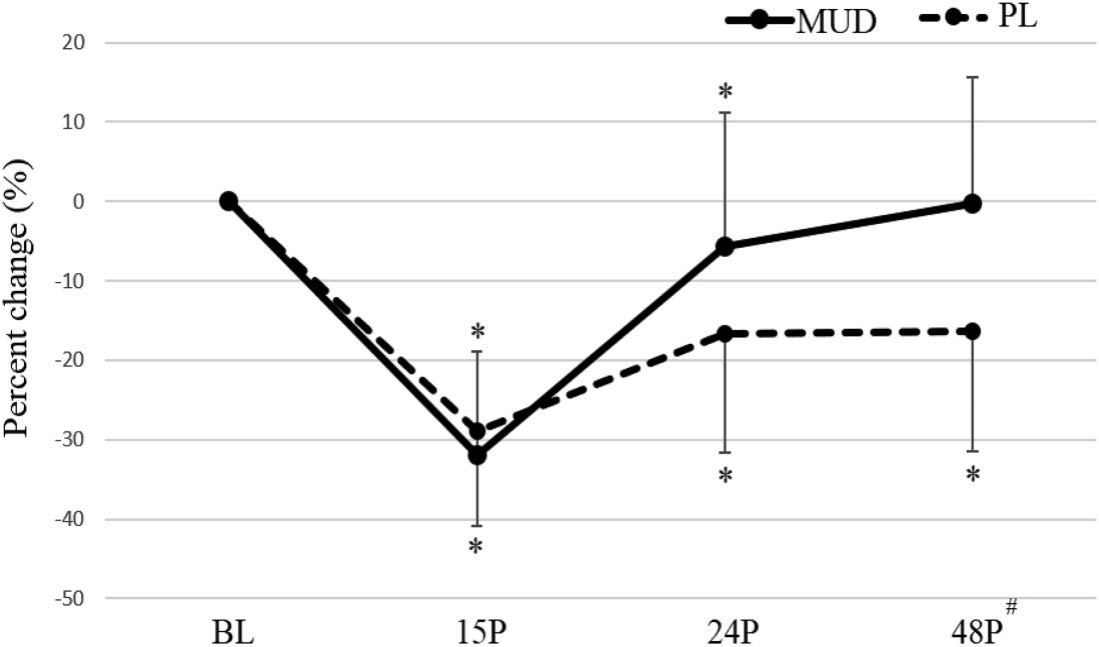
Percent changes in ISOK25 in MUD and PL at the different timepoints. ISOK25 = isokinetic leg press at 25 cm·s^−1^; MUD = menthol and *Arnica montana* mud pack; PL = placebo. #Indicates a significant (*p* ≤ 0.05) difference between the trials. *Indicates a significant (*p* ≤ 0.05) difference from BL. Error bars represent *SD*.

All the results of performance assessments are reported in Table 1. A significant trial × time interaction was found for ISOK25 (*F* = 4.936; *p* = 0.022; η^2^ = 0.414). A significant trial difference in ISOK25 was observed at 48P (*p* = 0.018) (Figure 2). For PL, ISOK25 was significantly reduced from BL at 15P (−28.9%; *p* = 0.003; confidence interval [CI]: 24.23–95.39), 24P (−16.7%; *p* = 0.008; CI: 10.26–59.62) and 48P (−16.4%; *p* = 0.005; CI: 12.16–57.56), whereas for MUD, ISOK25 was reduced from BL at 15P (−31.9%; *p* = 0.001; CI: 33.53–98.43) and at 24P (−5.7%; *p* = 0.026; CI: 1.34–22.34) only.

**Table 1:** Results of the assessments of performance, muscle architecture of VL and muscle soreness.*†

Assessment▼	Timepoint►	BL	15P	24P	48P
	Trial ▼				
CMJP	MUD	45.6 ± 7.9	36.3 ± 5.4‡	43.7 ± 6.3	45.4 ± 7.5
	PL	45.4 ± 9.6	35.5 ± 8.1‡	42.3 ± 7.6	43.7 ± 8.4
ISOK75 (N)	MUD	145.4 ± 21.6	112.7 ± 21.5‡	137.7 ± 21.3	145.1 ± 28.2
	PL	138.5 ± 25.6	102.9 ± 38.3‡	128.9 ± 28.3	139.8 ± 28.7
ISOK25 (N)	MUD	207.0 ± 35.7	141.0 ± 27.1‡	195.2 ± 37.7‡	206.4 ± 39.6
	PL	211.5 ± 33.2	151.7 ± 25.6‡	176.1 ± 32.3‡	176.2 ± 40.4‡§
ISQ (N)	MUD	2,233.1 ± 356.2	1790.5 ± 365.6‡	2,185.7 ± 411.1	2,307.1 ± 380.3
	PL	2,242.2 ± 387.2	1855.4 ± 349.7‡	2,184.0 ± 461.2	2,192.5 ± 431.5
VLMT (mm)	MUD	1.91 ± 0.31‡	2.25 ± 0.28‡	2.02 ± 0.27‡	1.97 ± 0.29‡
	PL	1.88 ± 0.27‡	2.22 ± 0.32‡	2.01 ± 0.26‡	1.93 ± 0.25‡
VAS (mm)	MUD	0	64.0 ± 24.6‡	29.4 ± 18.2‡	15.3 ± 13.0‡
	PL	0	66.5 ± 25.6‡	61.7 ± 21.1‡§	42.4 ± 28.7‡§

* MUD = menthol and *Arnica montana* mud pack; PL = placebo. CMJP = countermovement jump power; ISOK75 = isokinetic leg press at 75 cm·s^−1^; ISOK25 = isokinetic leg press at 25 cm·s^−1^; VLMT = vastus lateralis muscle thickness; VAS = visual analog scale.

† Data are reported as mean ± *SD*.

‡ Indicates a significant (*p* ≤ 0.05) difference from BL.

§ Indicates a significant (*p* ≤ 0.05) difference between the trials.

No significant trial × time interactions were observed for CMJP (*F* = 0.427; *p* = 0.598; η^2^ = 0.051), ISOK75 (*F* = 0.407; *p* = 0.738; η^2^ = 0.55), and ISQ (*F* = 2.070; *p* = 0.151; η^2^ = 0.206). Significant main effects of time were observed for CMJP (*F* = 31.981; *p* < 0.001; η^2^ = 0.051), ISOK25 (*F* = 36.009; *p* < 0.001; η^2^ = 0.837), ISOK75 (*F* = 55.011; *p* < 0.001; η^2^ = 0.887), and ISQ (*F* = 43.189; *p* < 0.001; η^2^ = 0.844). With trials combined, a significant change from BL was detected for CMJP (*p* < 0.001; CI: 4.31–14.92) and for ISQ (*p* < 0.001; CI: 248.14–581.19) at 15P only.

### Ultrasound Measurements

**Figure 3.**
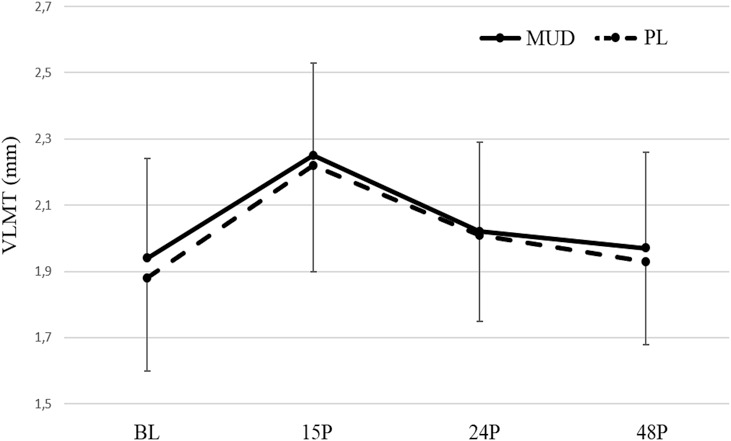
Changes in VLMT in MUD and PL at the different timepoints. VLMT = muscle thickness of vastus lateralis. MUD = menthol and *Arnica montana* mud pack; PL = placebo. #Indicates a significant (*p* ≤ 0.05) difference between the trials. *Indicates a significant (*p* ≤ 0.05) difference from BL. Error bars represent *SD*.

Changes in all ultrasound measures are presented in Table 1 and Figure 3. No significant trial × time interactions were found for VLMT (*F* = 2.286; *p* = 0.131; η^2^ = 0.246). Significant main effects of time, however, were noted for VLMT (*F* = 4.582; *p* = 0.032; η^2^ = 0.294). With trials combined, significant changes from BL were detected at 15P (*p* < 0.001; CI: −0.46 to −0.21), 24P (*p* = 0.002; CI: −0.18 to −0.5), and 48P (*p* = 0.006; CI: −0.11 to −0.02).

### Soreness Measurements

**Figure 4.**
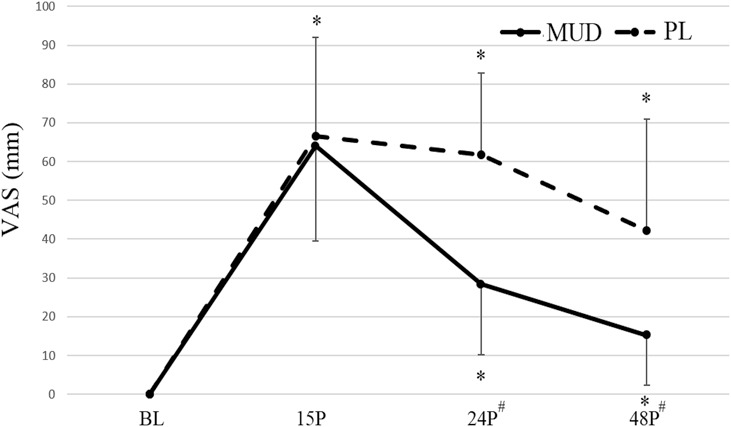
Muscle soreness of quadriceps muscle in MUD and Pl at the different timepoints. VAS = visual analog scale. MUD = menthol and *Arnica montana* mud pack; PL = placebo. # Indicates a significant (*p* ≤ 0.05) difference between the trials. *Indicates a significant (*p* ≤ 0.05) difference from BL. Error bars represent *SD*.

The results of the VAS for soreness can be observed in Table 1 and Figure 4. A significant trial × time interaction (*F* = 11.377; *p* = 0.001; η^2^ = 0.587) was observed for soreness intensity. Pairwise comparisons revealed that subject soreness intensity was elevated from BL following both trials; the soreness intensity following PL was significantly greater than MUD at 24P (*p* = 0.002), and 48P (*p* = 0.015).

## Discussion

The main aim of the present investigation was to assess the influence of mud packs including menthol and *Arnica montana* on the recovery process following a high-volume resistance training session focused on lower-body muscles. The results showed that significant reductions in power performance and maximal force occurred after the examined high-volume workout for the lower-body, and were still persistent 48 hours after the training session. In addition, significant changes in muscle architecture were detected 48 hours after the resistance training session. This finding is consistent with other studies that reported significant drops in strength and power performances and significant changes in muscle morphology still present 72 hours after a high-volume exercise protocol for the lower body (2,17). In addition, the results showed that MUD significantly enhanced the recovery rate following a high-volume resistance training session. In particular, the force expressed at the isokinetic leg press at 25 cm·s^−1^ (ISOK25) was restored within 48 hours after workout in MUD, whereas this parameter was still reduced at 48P in PL. ISOK75 and ISQ do not seem to be influenced by MUD. The specificity principle in resistance training implies that greater gains in performance are obtained at the specific training velocity (28). The same principle may suggest that acute exercise-induced drops in performance may be greater when tested at the specific exercise speed. In ISOK25 indeed, a greater drop in performance (−30.5%) was registered at 15P compared with both ISOK75 (−24.0%) and ISQ (−18.5%) at the same time point. Squat and leg extension were characterized by a 2-second time for both the eccentric and the concentric phases and the movement speed may be similar to ISOK25. Mud packs used in the present investigation consisted of a blend of essential oils, including a 5% of menthol and a 3% of *Arnica montana*. The enhanced recovery rate of muscle strength in MUD compared to PL may be related to the effect of menthol on neuromuscular function. A study by Topp et al. (33) reported significant reduction in popliteal blood flow following a bout of leg muscles contractions, using a 3.5% menthol gel. Reduction in blood flow may be related to the activation of thermosensitive neurons and adrenergic receptors (32). This effect may improve the recovery process and reduce the perception of pain following training sessions (23). This is supported by a study of Gillis et al. (16) that found a significant positive effect of topical menthol in preserving lower body power following a repeated sprint protocol. In this study, a 4% menthol gel applied twice a day, reduced the drop in vertical jump performance in the 96 hours after a muscle damaging exercise protocol, compared with a placebo.

Significant changes in muscle architecture from BL were detected 15 minutes after the lower-body damaging protocol and the initial condition was not restored within 48 hours in both MUD and PL protocols. Muscle swelling, indeed, does not seem to be influenced by the application of topical products. Changes in muscle architecture following high-volume resistance training protocols are related to vasodilation (34), reactive hyperemia, and delayed onset of muscle swelling (9). Muscle swelling is also related to the inflammatory response after high-volume resistance exercise protocols (10). Previous investigations (3) reported significant positive effects of active recovery on the recovery process following a damaging exercise session for the upper body. The effects, involving performance and muscle morphology, were attributed to transitory increases in local blood flow and temperature induced by active recovery (9). These transitory effects may represent a stimulus to muscle recovery influencing muscle inflammation after a damaging resistance exercise protocol. The vasoconstrictor effect of menthol may not be appropriate to reduce exercise-induced muscle swelling. Results of the present investigation also showed a significant effect of MUD on muscle soreness. A growing body of research has explored menthol's acute influence on postexercise delayed onset of muscle soreness. Johar et al. (23) indeed, reported that a menthol based topical gel was more effective than ice in reducing postexercise soreness.

Despite the fact that *Arnica montana* is one of the most popular treatment in complementary medicine (21), to date its efficacy has not been clinically supported by placebo-controlled trials (12,19). The beneficial effects of mud packs are often associated with their thermal therapeutic and heat conduction properties. In the present investigation, both MUD and PL, however, were applied at ambient temperature. Some studies suggest that a specific action of organic and inorganic components of mud should also be considered (27). More research, however, is needed to demonstrate the efficacy of mud packs therapy at ambient temperature on pain and inflammation. A possible limitation of the present investigation may be represented by the different smells and the cooling sensations on the skin surface of MUD and PL. However, in this study, no camphor or menthol were included in the PL composition to prevent any possible effect of these ingredients on the recovery process (16).

**Practical Applications**

The results of the present study suggest that mud packs with menthol and *Arnica montana* may accelerate the recovery process and reduce muscle soreness following a damaging resistance training workout for lower body. Strength and conditioning coaches may consider to use this strategy to enhance recovery following high-volume resistance training sessions. Mud packs may be particularly indicated when high demanding resistance workouts or competitions are performed in a short time and recovery is crucial for athletic performance.
